# Birth location preferences of mothers and fathers in rural Ghana: Implications for pregnancy, labor and birth outcomes

**DOI:** 10.1186/s12884-015-0604-2

**Published:** 2015-08-12

**Authors:** Leslie E. Cofie, Clare Barrington, Kavita Singh, Sodzi Sodzi-Tettey, Akalpa Akaligaung

**Affiliations:** Department of Health Behavior, University of North Carolina, Gillings School of Global Public Health, 302 Rosenau Hall, CB #7440, Chapel Hill, NC 27599-7440 USA; Carolina Population Center, UNC-Chapel Hill, Chapel Hill, NC 27599-7445 USA; Department of Maternal and Child Health, University of North Carolina, Gillings School of Global Public Health, 401 Rosenau Hall, CB #7445, Chapel Hill, NC 27599-7445 USA; Project Fives Alive!/Institute for Healthcare Improvement, Accra, Ghana; Boston University School of Public Health, 15 Albany St, Boston, MA 02118 USA

**Keywords:** Maternal health, Health facility birth, Homebirth, Birth location preference, Ghana

## Abstract

**Background:**

Maternal deaths in Sub-Saharan Africa are largely preventable with health facility delivery assisted by skilled birth attendants. Examining associations of birth location preferences on pregnant women’s experiences is important to understanding delays in care seeking in the event of complications. We explored the influence of birth location preference on women’s pregnancy, labor and birth outcomes.

**Methods:**

A qualitative study conducted in rural Ghana consisted of birth narratives of mothers (*n* = 20) who experienced pregnancy/labor complications, and fathers (*n* = 18) whose partners experienced such complications in their last pregnancy. All but two women in our sample delivered in a health facility due to complications. We developed narrative summaries of each interview and iteratively coded the interviews. We then analyzed the data through coding summaries and developed analytic matrices from coded transcripts.

**Results:**

Birth delivery location preferences were split for mothers (home delivery–9; facility delivery–11), and fathers (home delivery–7; facility delivery–11). We identified two patterns of preferences and birth outcomes: 1) preference for homebirth that resulted in delayed care seeking and was likely associated with several cases of stillbirths and postpartum morbidities; 2) Preference for health facility birth that resulted in early care seeking, and possibly enabled women to avoid adverse effects of birth complications.

**Conclusion:**

Safe pregnancy and childbirth interventions should be tailored to the birth location preferences of mothers and fathers, and should include education on the development of birth preparedness plans to access timely delivery related care. Improving access to and the quality of care at health facilities will also be crucial to facilitating use of facility-based delivery care in rural Ghana.

## Background

Approximately half of the world’s maternal deaths occur in Sub-Saharan Africa, mostly as a result of complications including hemorrhage, eclampsia, obstructed labor, and infections [1–3]. Many of these deaths would be largely prevented with health facility delivery assisted by skilled birth attendants (SBAs) [4, 5]. Yet, a large percentage of women in West Africa do not deliver in health facilities [3, 6]. In Ghana, 68 % of births occur with the assistance of a SBA and 67.4 % of births occur in health facilities [7]. Just over half of all births (52.7 %) in rural areas occur in facilities [7]. Also, facility delivery is lowest in the Northern (37 %) and Central (61 %) regions of the country [7].

**Fig. 1:**
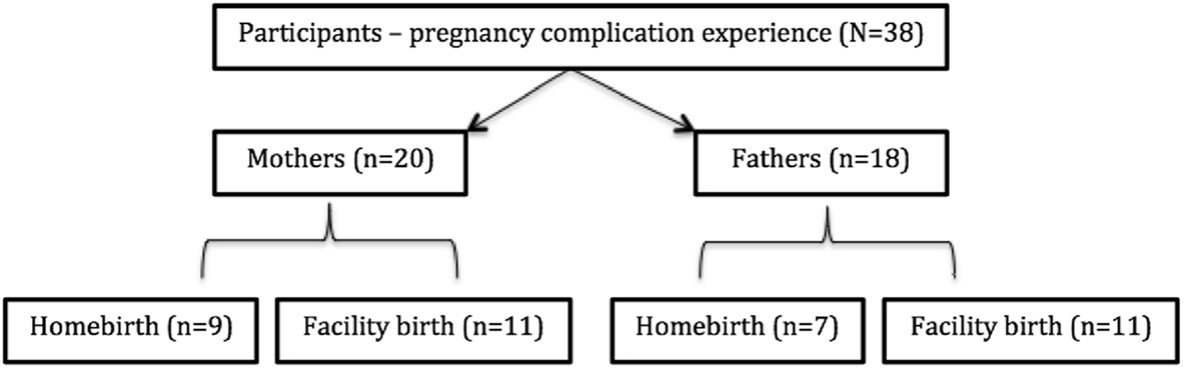
Birth location preferences. Number of mothers and fathers who preferred health facility birth compared with those who preferred homebirth

In low- and middle-income countries (LMICs) like Ghana pregnant women living in rural areas may fail to seek health facility delivery care until the onset of labor complications, thus presenting in facilities too late to be helped [8–10]. Factors associated with facility delivery use have been conceptually examined in previous reviews [11–13]. One study’s framework presented three categories of factors related with health facility delivery: physical and economic accessibility, sociocultural, and perceived needs and benefits of facility delivery [13]. For example, it determined that facility delivery was related to factors including long distance from and high cost of health facility care, maternal socio-demographic characteristics (e.g. education, age, autonomy), poor quality of care, and previous use of antenatal care.

A few subsequent reviews focused on the sub-Saharan African Region [14–16]. Most recently, a systematic review of determinants and barriers to health facility delivery in sub-Saharan Africa defined five categories of factors influencing facility delivery: maternal, social, antenatal care (ANC), facility related, and macro-level factors [14]. Notably, social factors such as attitudes about the benefits of health facility births and macro-level factors like government subsidy of health care were identified as determinants of facility delivery use. In the case of Ghana, cost exemption from the National Health Insurance Scheme and free maternity care including delivery in health facilities for pregnant women have contributed to uptake in health facility delivery [17, 18].

To better understand how to address delays in the use of skilled delivery, numerous studies have examined determinants of whether a woman delivers in a health facility or at home. These studies suggest that reasons for health facility delivery include accessibility, good perceptions about the safety of health facility births, positive attitudes towards health providers and quality of care received for facility births [15, 19, 20]. Conversely, reasons for homebirths include the cost-effectiveness and convenience of homebirths, having family members nearby during women’s labor experiences, and maintaining traditional childbirth and postpartum practices [19–22]. Additional reasons for homebirths among pregnant women include social pressure from family members, social norms, and past experiences of using traditional birth attendants (TBAs) [23, 24].

Birth location preferences in LMICs have mostly been examined among women, while research on fathers’ preferences is lacking. Some studies have examined men’s role and involvement in the birth preparedness planning and maternal health services use of their spouse/partners [25–27]. Recent work focusing on perspectives of fathers indicated that they were generally supportive and encouraging of their partners’ use of health services [28–30]. Since they influence the type of care their spouse/partners receive during pregnancy and labor, there is a need to further understand father’s birth location preferences [31].

Another gap in the existing literature is the influence of birth location preferences on pregnancy, labor and birth outcomes. Exploring how birth location preferences may influence pregnant women’s health-seeking experiences is critical, as these experiences likely contribute to delays in seeking care until onset of birth complications and ultimately impacts birth outcomes [32]. Understanding how preferences could potentially impact birth outcomes can help inform the development of appropriate health promotion and community-based strategies to facilitate birth preparedness, and further improve maternal and newborn survival.

To address the aforementioned gaps we examine the birth location preferences of mothers who experienced pregnancy/labor complications, and fathers whose partners experienced such complications in their last pregnancy. Our study’s objective is to explore how birth location preferences influenced women’s pregnancy and labor experiences, and the subsequent impact on these experiences on their birth outcomes. Research has pointed out the need to examine birth location preferences within specific local contexts [9, 12, 13]. To that end, this study is based on the experiences of mothers and fathers in two regions of rural Ghana.

## Methods

### Design & sample

The present study is based on a baseline qualitative assessment of barriers faced by pregnant women in accessing health care services during pregnancy and delivery in Ghana to inform a community-level quality improvement intervention to promote maternal and newborn health services access and utilization. We collected data in two districts, one in the Northern Region (NR) and the other in the Central Region (CR) between May and June of 2012. We purposively sampled mothers (*n* = 20) who experienced pregnancy or labor complications themselves or whose newborns experienced complications, and fathers (*n* = 18) whose wives/partners experienced such complications. The fathers and mothers sampled were not partners. Complications included severe ailments experienced by women or newborns (e.g. severe bleeding, infections, or obstructed labor), which resulted in an urgent visit to a health facility. As an inclusion criteria women, or their newborns, had to have been referred from a community-level health post to a health center, or from the health center to a high-level facility like a hospital in the last year. This was in order to ensure that participants selected had experience with pregnancy complications. Nearly all women in our sample delivered in health facilities due to complications; the two women who experienced homebirths sought facility care for postnatal complications. Additional criteria were age 18 years or older, and natives of the Northern and Central Region.

Health workers from local health centers generated a list of women who experienced complications. Based on this list, we worked with the assistance of community health workers/ local assemblymen to identify mothers and fathers in communities across the two districts. We visited the households of women and husbands/ male partners of women who met the inclusion criteria. We interviewed those who agreed to participate, and the age range of the participants was 18–45. Participants from the Northern region were of Konkomba or Nanumba ethnicity. The Konkomba people are either Christians or traditionalists, and the Nanumbas are mostly Moslems. Participants in the Central region were of the Fante ethnicity and predominantly Christian. A total of 38 birth narrative interviews were conducted, following the principle of data saturation – i.e. the point at which collecting more data did not yield new information or themes related to our research study [33].

### Data collection

We developed a semi-structured interview guide based on evidence from the literature and multiple reviews from the research team. A male and female Ghanaian research assistant (RA) in each study region, fluent in the local languages of the regions, underwent a two-week training on conducting field interviews. The interview guide was field tested before final revisions were made. The male and female RAs interviewed male and female participants, respectively. Participants were asked to describe pregnancy and labor experiences, use of health services during pregnancy and labor, birth delivery preferences and plans and support received during pregnancy. Sample questions included the following: 1) Describe what you remember about your pregnancy experience, labor and delivery experience. 2) During your pregnancy did you have a place in mind you preferred to give birth? 3) What were reasons for your choice of birth delivery place? 4) What care did you receive for your pregnancy?

Verbal informed consent was obtained from all study participants. The interviews lasted for about an hour each, and participants were provided with bars of soaps as an appreciation for their participation. RAs conducted interviews in two local Ghanaian languages (*Twi* and *Dagbani*). The interviews were audio recorded, transcribed, and translated to English. We obtained ethics review approval from the Ghana Health Service Ethical Review Committee and the University of North Carolina at Chapel Hill Institutional Review Board.

### Data analysis

Following data collection we conducted close readings of all birth narrative interviews and wrote narrative summaries on each participant’s birth experience. Based on these summaries, we generated preliminary descriptive codes and memos of participants’ birth preferences. Then, through discussion of emergent findings with the research team including the local Ghanaian PI, and subsequent review of the transcripts, we developed a core set of codes in order to conduct thematic analysis. The first author applied these codes to the birth narratives using Atlas.ti software (version 7.0, Scientific Software Development GmbH, Eden Prairie, MN), during which the initial coding scheme was modified and additional codes were added. We then reviewed code outputs and developed code summaries and analytic matrices [34, 35]. The code summaries provided contextual information on health seeking experiences that resulted in women’s birth outcomes. The matrices enabled comparison between participants with home versus facility birth preferences on their reasons for birth location preferences, pregnancy and labor experiences, and resulting birth outcomes.

## Results

Among the 38 participants interviewed preference for homebirths and health facility births were split among mothers (home delivery–9; facility delivery–11) and more fathers preferred facility over home delivery (home delivery–7; facility delivery–11) (Fig. 1). For each preference category we first describe factors attributed to birth location preference. We then present how such preferences may have influenced women’s health-seeking behaviors and experiences during pregnancy and labor, and subsequent birth outcomes.

### Homebirth preference

Participants preferred homebirths due to normative perceptions, previous experience of homebirths, high costs of traveling to health facilities, and distance to such facilities. Participants who described homebirths as normative perceived such practice as existing as far back as they remembered. One young father [Farmer, 20 yrs., NR] preferred homebirths because he was born at home. An older father elaborated:

*…we live in a village and because of that we always want our women, if not because of things beyond our control, to deliver at home… Yes we wish it [homebirths] very much, if it were so simple, we would have preferred that* [Farmer, 60 yrs., NR].

This reflects homebirth preference as a norm in the village that spans generations, a view supported by several mothers as well.

Mothers who preferred home births also echoed the idea in the quote above that the ability to deliver at home is beyond a pregnant woman’s control. They noted that while they try to give birth at home, they are willing to accept “*whatever God will give*” them, since labor complications may result in them having to seek care at a health facility. Acknowledging the unpredictable nature of women’s labor experiences, health facility delivery care was considered a last resort, once it became clear that the attempt to deliver at home was not feasible.

Other mothers and fathers preferred homebirths based on past successful experiences with homebirths. For example, two fathers mentioned that they preferred homebirths because their wives’ previous births, from eight and six pregnancies respectively, were homebirths. Participants generally associated previous homebirths with relatively manageable birth experiences. For example, one mother with a homebirth preference explained:

*When you deliver in the house, they will say your delivery wasn’t challenging…Some [women] when they are to deliver they have complication… some [women] too, when they feel pains, you will see that they walk a little, you see that they will just come and deliver* [Farmer, 20 yr., NR].

This mother’s comments reflect the view of other mothers who, looking back on their previous birth experiences, associated homebirths with having an easy labor experience.

A number of participants alluded to costs associated with traveling to health facilities, as being reasons for preferring homebirths. In getting to such health facilities, pregnant women often had to endure traveling through poor road conditions. For example, a father (Farmer, 35 yrs., NR) who preferred homebirth delivery argued,

“*The reason being that our roads are not good and so it is problematic to travel with them [pregnant women] on such roads, that is not good for their health.*”

The combined effect of poor roads and use of an unsuitable but common means of transportation like motorcycles was considered detrimental to women’s health and was a reason to prefer delivering at home among both men and women.

Participants whose homebirth preference was due to concerns regarding distance and cost associated with facility-based care, tended to avoid health facility care until the onset of complications. Once women’s health conditions became critical, their relatives were forced to borrow motorcycles and pay for fuel, or pay for a vehicle to transport the women to a health facility. Some pregnant women had to be carried on bicycles and some walked to the facilities. A lack of birth preparedness or an emergency plan among participants provides a striking example of how homebirth preferences may have contributed to women’s experiences, including severe complications for women and babies and even neonatal death.

For instance, a mother with a preference for homebirths (Farmer, 20 yrs. NR) sought the assistance of a TBA during a prolonged labor. The mother was eventually sent to a health center to receive further birth assistance, as the TBA’s efforts were unsuccessful. She later mentioned, “*The child died in my stomach before they even operated*.” Her preference for home delivery delayed her seeking of facility care. We also observed three similar narratives of prolonged labor among women with a preference for homebirth. The women mentioned that they lost their newborns during birth, and some experienced postpartum illness. According to their narratives they spent substantial time attempting to achieve home birth delivery, by initially seeking the assistance of TBAs during labor, and relying on traditional medicine. Their use of health facilities was a last alternative for receiving treatment for complications once it became evident that homebirth was unachievable. The women’s experiences compounded the challenges in receiving care in facilities – e.g. distance, cost, and quality of care – and possibly contributed to their negative health outcomes.

### Health facility birth preference

Participants who preferred health facility delivery expressed concerns about risks involved in homebirths, the need for skilled care for birth complications, and personal concerns about pregnant women’s health, as explanations for their preference. Additional reasons included previous experience of facility birth and the perceived high prevalence of, or a shift in norms towards, facility delivery in certain communities.

Certain participants preferred health facility births because of their awareness of health risks associated with childbirth. In one mother’s [Farmer, 30 yrs., NR] view, delivering at the clinic was safer, since she and her baby would receive care in case of any complications. A father with the same opinion maintained that he was mainly concerned with finding the right kind of health facility where his wife could experience safe delivery.

A few participants described personal health concerns that prompted their intentions to seek facility-based birth delivery. Notably, one mother explained, “*Because I am not well, whatever happens, I will go to the hospital to give birth because I don’t know what will happen to me”* [Farmer, 45 yrs., NR]. She experienced diarrhea and vomiting during pregnancy. She sought care at a health center and was then referred to a main hospital where she received the appropriate care for her ailment. This mother’s narrative parallels that of a young father (Farmer, 20 yrs., NR) who described his wife’s pregnancy experience as very concerning. According to him his wife had difficulties sleeping at night. Besides her regular attendance to the health facility for antenatal care, he had to “*carry her to the hospital*” a few times because of her illness. Consequently, to avoid labor complications, the father and his wife planned for her to deliver at a health facility.

Similar to the homebirth preference, health facility delivery preference for some participants was based on whether women’s previous pregnancies resulted in facility births. For instance, one mother (Hairdresser, 28 yrs., CR) preferred to give birth at her community’s health facility because her two children were born there. She expressed her satisfaction with the role played by health providers in assisting with her previous delivery:

*The midwife here is good and she does not scream at people in labour. As you know, going to deliver is a very painful thing and some of the midwives scream or shout at pregnant woman in labour. [Hairdresser, 28 yrs.]*

Several participants who utilized health centers staffed by helpful and caring midwives shared this mother’s sentiments about supportive care from the SBA. Some also noted that the midwives often accompanied pregnant women in labor to the referral hospital and made visits to the women’s home postpartum. This was not always the case, as other participants who preferred homebirths indicated that negligent SBAs staffed certain health centers. As evidence, one mother provided the following explanation:

*I was also crying when I saw and heard my child crying. One of the nurses came to insult me that "what is the meaning of the tears that I am shedding over there?" I asked her why she was asking me the question; could she not see my child crying? She did not mind me but she just left the room [Occupation unknown, 34 yrs., CR].*

The mother’s baby was crying because it was not being attended to, which caused her to cry too. Her experience with the nurse indicated that the nurse was both rude and unhelpful, as she refused to assist the mother with her crying child.

Two fathers perceived childbirth at the health facility in their community as a regular practice. According to them, it is expected that women would give birth at that facility. One of the fathers provided further insight into why community members were seeking facility skilled delivery:

*She [his wife] always gives birth at the hospital. So, she was always going to hospital as scheduled for her during the pregnancy. Also, if one is pregnant and you do not go to the hospital, the staffs over there may refuse to take care of you when you go into labour* [Farmer, 50 yrs., NR]

Community members like the participant’s wife sought antenatal care throughout their pregnancy term at their local health facility, in part because they perceived failure to do so may result in denial of care from SBA during labor.

The general narrative of participants who preferred facility births indicated that besides seeking health facility care for pregnancy/labor complications, women were overall likely to use facility care throughout their pregnancy experience. That is, their preference of facility delivery likely led them to access health facilities more frequently than participants who preferred homebirth. Despite having to overcome common barriers to seeking health services in rural settings, we found that almost all of the women successfully delivered their infants following referral to health facilities. The two exceptions were those who sought care at the same health center noted for being of poor quality and having uncaring staff members.

For example, a father (Famer, 37 yrs., CR) who preferred homebirth described his wife’s attempt to seek care at that facility when she was in labor as distressful. His account of this experience revealed that despite seeking care at the health center, her labor was prolonged. His wife was turned away and told to seek care at a hospital farther from their community because the health workers were attending a party. She subsequently experienced a stillbirth. The father’s narrative highlights the notion that besides one’s preference for and effort to achieve facility delivery, other factors related to delay in seeking care (e.g. lack of provider support) are beyond the control of an individual.

Ultimately, this group of participants’ birth location preference enabled them to decide on a plan for receiving care from a SBA once pregnant women went into labor. Generally, their birth narratives showed that the course of action for when a pregnant woman went into labor or experienced complications was to initially seek care at a health facility. Such initiative likely allowed them to avoid some of the dangers associated with prolonging needed care for labor complications at a health facility.

## Discussion

Many homebirths in LMICs, especially in rural areas, are assisted by TBAs who are usually more readily accessible than SBAs at a health facility [10, 36]. Access to health facility for delivery care is more challenging and thus requires pregnant women and their families to plan in advance to seek health care. Among study participants, we observed two patterns of birth location preferences and outcomes among participants. The first, homebirth preference, appeared related with several cases of stillbirths and postpartum morbidities, whereas the second, health facility birth preference, was associated with early care seeking that likely enabled women to avoid adverse effects of prolonged labor and birth complications.

Our finding that homebirth is a socially accepted norm is supported by literature from other LMICs [10, 19]. Previous studies in certain rural areas of Ghana suggested that homebirths were highly valued and considered the “ideal situation,” as it meant normal delivery, convenience and increase of a woman’s social status [37, 38]. In other studies women considered health facility-birth unnecessary, as most women had experienced successful homebirths [19, 39, 40]. Also, as we found, cost and distance associated with health facility delivery served as additional deterrents from sending otherwise normal pregnancies for facility births [10, 41, 42]. Study participants who preferred homebirth, generally lacked a birth preparedness strategy to seek health facility care for pregnancy complications. Research has shown that pregnant women with a birth preparedness plan compared with those without one were more likely to deliver in health facilities [43]. In our study the lack of a plan among the homebirth preference group likely contributed to delays in seeking facility care for pregnancy related complications.

Also our findings suggest that participants who preferred health facility delivery seemed more prepared and readier to engage in the health-seeking process compared with those with homebirth preference. This is evidenced by the former’s efforts to seek facility delivery at first sign of labor. Reasons for preferring health-facility delivery are corroborated by previous findings [12, 13]. For example, a recent review of determinants of facility delivery in sub-Saharan African countries, linked facility births with factors such as concerns about pregnancy related complications, previous experiences of facility births, and desire for a SBA in case of birth complications [14]. These reasons stand in contrast to evidence indicating facility-level barriers to facility delivery include negative experiences of pregnant women.

Similar to our findings, previous work in Ghana has shown that uncaring attitude of health workers, mistreatment of pregnant women, poor quality of care and delays in receiving care deter women from seeking facility delivery [44]. In spite of these barriers recent work indicates a shifting norm in birth location preference from home to health facilities [45]. Family members appear to advocate health facility birth to prevent or address birth complications [9, 38, 46]. We even observed that some husbands were accompanying their wives to receive antenatal care at health facilities. Even so, challenges to facility delivery in a LMIC like Ghana persist in the form of limited resources in certain facilities, shortage of health workers including midwives, and cost (e.g. medication, supplies etc.) incurred by clients who utilize facility care.

As our study focused on women who experienced birth related complications, all participants had to contend with well-known factors that influenced care-seeking such as transportation, cost, support, availability, access, and quality of care [14, 36, 43]. Those with a preference for facility delivery tended to tackle these barriers prior to labor or at the onset of labor, whereas participants with preference for homebirths appeared less prepared and slower to take action beyond the home. Accordingly, we observed that the pregnancy and labor experiences of pregnant women who preferred homebirth likely contributed to adverse outcomes.

Having birth preparedness plans such as identifying a facility, SBA and transportation, and also saving money toward delivery costs can contribute to the reduction in delays in seeking facility-based care [32]. In determining how to encourage birth preparedness among pregnant women, it is important to ascertain their birth location preference early on. As our findings suggest, these preferences impact pregnancy-related care experiences of pregnant women, which has important implications for interventions to promote safe birth deliveries. For example, women with a preference for facility delivery may be receptive to developing a birth preparedness plan that increases their accessibility to such facility. However, those who prefer homebirths may require an intervention that focuses on safe homebirths with an added alternative of an effective referral process that could enable timely access to health facilities in cases of complications.

Inclusion of fathers in our study adds to a small body of work that examines men’s function in the reproductive health experiences of their partners [25–31]. We found that fathers had an influential role in their wives’ use of pregnancy and delivery care, such that their birth location preferences impacted the women’s health-seeking behaviours. For instance, fathers who preferred health facility births perceived the need to send their wives to a facility to receive treatment for pregnancy and labor-related complications. They did not consider facility care as their last resort. Other studies similarly suggested that pregnant women whose partners were involved in their pregnancy and delivery care were more likely to use health facility care services than men who were not involved in this care [28, 47, 48].

### Limitations

Our study is limited to narratives of pregnant women and fathers who experienced birth complications. Additional insights may be gleaned from pregnant women who have uncomplicated home or facility births. The experiences of participants were examined retrospectively. Hence, there is potential for bias in their recall, and their recent experiences may have influenced their perceptions about their birth location preferences. Future work should prospectively examine pregnant women’s experiences, and also include the perspective of health providers and key community members who are involved in care for pregnant women and newborns. Our sample was purposefully drawn from health centres in two rural areas of Ghana, and therefore may not be generalizable to other countries. However we do believe our findings may be transferable to other settings, such as other rural areas of LMICs that have traditionally experienced similar challenges to improving the use of health facility birth.

## Conclusion

Delay in seeking care, especially among pregnant women in rural areas, has long been a topic of concern for the global health community. While factors associated with this delay are well known, our study contributes to an understanding of the role of birth location preference as an influence on delaying delivery care and the implications of these preferences for birth outcomes. Efforts to promote safe pregnancy and childbirth must identify women’s’ birth location preference early on in order to tailor health education and interventions to their needs. Besides encouraging pregnant women who are already inclined to deliver at a health facility to develop detailed birth preparedness plans, improving access and quality to health facilities will be crucial to facilitating use of skilled delivery. Those who prefer home delivery should also be educated about having birth preparedness plan in case they experience unforeseen complications requiring skilled care. Health promotion strategies can focus on working with women at the community level to develop birth preparedness plans for situations when it would be necessary to gain access to health facility care.
